# Novel Features and Considerations for ERA and Regulation of Crops Produced by Genome Editing

**DOI:** 10.3389/fbioe.2018.00079

**Published:** 2018-06-18

**Authors:** Nina Duensing, Thorben Sprink, Wayne A. Parrott, Maria Fedorova, Martin A. Lema, Jeffrey D. Wolt, Detlef Bartsch

**Affiliations:** ^1^Bundesamt für Verbraucherschutz und Lebensmittelsicherheit, Berlin, Germany; ^2^Institute for Biosafety in Plant Biotechnology, Julius Kuehn Institute, Quedlinburg, Germany; ^3^Department of Crop and Soil Sciences, Institute of Plant Breeding, Genetics and Genomics, University of Georgia, Athens, GA, United States; ^4^Corteva Agriscience™, Agriculture Division of DowDuPont™, Johnston, IA, United States; ^5^Biotechnology Directorate, Ministry of Agro-Industry, Buenos Aires, Argentina; ^6^National University of Quilmes, Bernal, Argentina; ^7^Department of Agronomy and Crop Bioengineering Center, Iowa State University, Ames, IA, United States

**Keywords:** genome editing, environmental risk assessment (ERA), regulation, new breeding techniques (NBT), CRISPR/Cas, ISBR, ISBGMO

## Abstract

Genome editing describes a variety of molecular biology applications enabling targeted and precise alterations of the genomes of plants, animals and microorganisms. These rapidly developing techniques are likely to revolutionize the breeding of new crop varieties. Since genome editing can lead to the development of plants that could also have come into existence naturally or by conventional breeding techniques, there are strong arguments that these cases should not be classified as genetically modified organisms (GMOs) and be regulated no differently from conventionally bred crops. If a specific regulation would be regarded necessary, the application of genome editing for crop development may challenge risk assessment and post-market monitoring. In the session “Plant genome editing—any novel features to consider for ERA and regulation?” held at the 14th ISBGMO, scientists from various disciplines as well as regulators, risk assessors and potential users of the new technologies were brought together for a knowledge-based discussion to identify knowledge gaps and analyze scenarios for the introduction of genome-edited crops into the environment. It was aimed to enable an open exchange forum on the regulatory approaches, ethical aspects and decision-making considerations.

## Introduction

New plant breeding techniques, such as genome editing, enable a previously unachievable targeted and precise modification of the genome. They allow for the introduction of very precise genomic changes, ranging from the exchange, insertion or deletion of one nucleotide at one specific locus to the site-specific integration of entire genes. Genome editing comprises protein mediated techniques (e.g., TALENs, zinc-finger nucleases), nucleic-acid-mediated genome modifications (e.g., ODM), or a combination thereof (e.g., CRISPR-techniques). Molecularly, in most cases a DNA double strand break (DSB) is induced which is subsequently repaired by one of the endogenous cell repair mechanisms, non-homologous end joining (NHEJ) or homologous recombination (HR). The preferential repair mechanism in plants is NHEJ, which is prone to errors. Due to these errors small changes in the nucleotide sequence (mutations) can be induced at the repaired locus (Hsu et al., 2014; Bortesi and Fischer, 2015) which may result in variants useful to crop improvement.

On-going development and innovation in genome editing promise to increase its value as a tool for crop improvement. For instance, a modified Cas-nuclease allows the precise editing of target bases in genomic DNA without relying on double-strand breakage (Komor et al., 2016). In multi-target approaches, site-directed mutagenesis of several target genes can be tackled simultaneously (Svitashev et al., 2015; Chilcoat et al., 2017; Shen et al., 2017), and targeted transgene insertions at one specific locus (Ainley et al., 2013) could lead to trait stacking possibilities with yet unknown dimensions. All these developments occurred within just the last few years, and rapid progress is to be expected (Puchta, 2017).

Those genome editing applications that do not aim at the insertion of foreign genes, but at inducing site-specific mutations at single loci of a plant's own genetic material, are able to create organisms that could have come into existence naturally or through conventional breeding. Thus, although few regulators in some countries have instituted mechanisms for addressing the regulatory status of crops derived from genome editing (Whelan and Lema, 2015; Wolt et al., 2016), decisions as to whether or not they require legal regulation lag behind in most countries.

In the EU, it is still unclear whether edited organisms will fall under the Genetic Engineering Law. The court cases on CIBUS™ canola are pending in Germany, and no legal guidance has been published by the European Commission (for a summary see Sprink et al., 2016). Legal classifications of new plant breeding techniques, including various genome editing tools, were suggested by an independent EU member states expert team in 2011 (Lusser et al., 2011), but the report by a “New techniques Working Group” set up by the European Commission to assess whether or not plants created by certain breeding techniques fall within the scope of the genetically modified organism (GMO) legislation, which was finalized in 2011, has never been officially released (Kahrmann et al., 2017). The European Commission has not published any legal opinion on these techniques so far and is not expected to do so at short notice. During the hearing of October 3, 2017 in the Case C-528/16 at the European Court of Justice, the Commission vaguely stated that they were preparing something about this “new” problem. In contrast, according to its statement during that hearing, the Commission is of the opinion that mutagenesis is exempted from the Directive on deliberate release if no recombinant nucleic acid molecules are used.

And indeed, with the exception of Canada, regulatory authorities throughout the world do not consider mutagenesis as subject to regulation under biosafety laws. And even in Canada, traditional mutagenesis is not regulated unless it produces a novel trait. It is the novel trait that is regulated, not the method used to produce it.

In the USA, current decisions on genome-edited plants have been based on the Plant Protection Act, as enforced by the USDA. The US Coordinated Framework for Biotechnology makes no special provisions for genome edited crops. As for any biotechnology-derived plant, if the genome-edited crop poses a plant pest risk, expresses a pesticide trait, or poses food safety risks different from other plants produced through traditional plant breeding then it is subject to regulatory considerations (by USDA, EPA and FDA, respectively); otherwise, the product can freely enter market channels. A new regulatory framework for biotechnology that was drafted in the last 2 years was going to be based on a noxious weed designation, but it was withdrawn in late 2017 to re-engage with Stakeholders (USDA, 2017)^1^. USDA does not currently regulate, or have any plans to regulate plants that could otherwise have been developed through traditional breeding techniques as long as they do not pose a plant pest risk, that is, as long as they are developed without the use of a plant pest as the DNA donor or transformation vector and they are not themselves plant pests or noxious weeds (USDA, 2018)^2^. In addition, both the FDA and the EPA could regulate genome-edited crops, but neither agency has indicated what their approach will be.

Elsewhere, regulatory frameworks have been established that allow for progress in the development and commercial advancement of crops developed through genome editing, even as the specifics of the regulatory frameworks are being considered (Whelan and Lema, 2015; Wolt et al., 2016).

With the current lack of adequate legal guidance throughout much of the world, a debate has started whether the legal status of plants derived from genome editing has to be based on the process used to create the organism (process-based approach) or on the final product obtained by the process (product-based approach). Another point of discussion is whether point mutations created by genome editing techniques have the same legal status as point mutations created by spontaneous or by conventional induced mutagenesis. Moreover, in various countries, traceability requirements are in use to detect and to identify a GMO^3^. Yet, most point mutations—or even larger changes, as long as no foreign DNA is integrated into the final organism's genome—do not carry a tag displaying the technique used to create them. Finally, asynchronous or even contradictory regulation of organisms created by genome editing in different countries will disrupt world trade and collide with standards of the World Trade Organization (WTO).

Rapid progress in genome editing technologies is challenging risk assessment and post-market monitoring frameworks: Shall certain types of genome-edited crops pass a simplified procedure on risk assessment as was suggested by Huang et al. (2016)? Shall the types of genome edited crops that also could have been created by conventional breeding techniques (e.g., by classical mutagenesis) be regulated no differently from conventionally bred crops? Does the current scientific development represent the ultimate trigger to now design a novel framework for risk assessment which focusses on the product and its potential effects on health and environment irrespective of the technique used to develop it, as suggested by Conko et al. (2016)?

To help answer these questions, the session “Plant genome editing—any novel features to consider for ERA and regulation?” held at the 14th ISBGMO^4^, used both, expert presentations and an interactive “World Café” discussion to bring together scientists from various disciplines (molecular biology, modeling, genetics, ecology) as well as regulators, risk assessors and potential users of the new technologies. Recent technological developments were summarized and examples of current applications in plant breeding were collected. A science-based discussion was aimed at the identification of knowledge gaps and the analysis of scenarios for the introduction of selected edited organisms into the environment. The interactive session raised awareness of benefits and risks of the new techniques and provided the opportunity for an open exchange, connecting regulatory approaches, ethical aspects and decision-making.

### Key expert contributions on challenges, opportunities and perspectives of genome editing applications for crop plant breeding

Five talks were given in the session that provided a framework for the following discussion and “World Café.” Each talk focused on a different but major aspect or perspective relevant to the question at hand.

Risk assessment often starts by identifying hazards that are unique to the item being regulated. Accordingly, Wayne Parrott compared the result of genome editing with conventional plant breeding in an attempt to identify any new and unique features about genome editing:

The technology for genome editing developed very rapidly, and equally quickly found numerous applications across medicine and agriculture. The latter includes the modification of crop plant genomes. Due to the novelty of the technology, many groups are singling out edited plants as somehow being new and different from plant varieties produced in the past.

To properly address the question about what is unique, or at least new and different, about genome editing, it is first necessary to consider how new plant varieties are produced by conventional plant breeding. The changes that take place at the chromosome level during the breeding process are of particular relevance, as they serve a basis for comparison of the changes made by genome editing.

Modern row crop varieties or cultivars can be thought of as collections of various traits. These traits can affect the phenology of the plant, the quality of the product, or provide agronomically useful traits, such as resistance to abiotic stress, pests, and pathogens. Today, most seed catalogs will list all the relevant phenological traits and all the resistances found in any given cultivar or hybrid.

Each of these traits is the result of one or more genes. Usually, it is a matter of identifying the right allele of the gene, and breeding the desired allele into the cultivar. Aside from a few epigenetically controlled traits, all heritable traits reflect changes that take place at the DNA level (Weber et al., 2012; Schnell et al., 2015). Thus different alleles of a gene have different DNA sequences. The difference in the sequence can be as small as the substitution of a single base pair, or can consist of base pair deletions or insertions that range from single base pairs to thousands of base pairs.

A plant breeder will first search for desired alleles in other varieties of the same crop, or in its landraces or wild relatives (Acquaah, 2012). Failing that, a breeder may try to bring out variation through mutagenesis (Ahloowalia et al., 2004). The changes made by mutagenesis to the DNA range from single base pair substitutions, to inversions, insertions, and deletions of various sizes (Anderson et al., 2016).

Another alternative for breeders is to find the desired trait in a related species. Specialized laboratory procedures may be necessary to make the cross, but the practice has been on-going for the past century (Jones et al., 1995; Hajjar and Hodgkin, 2007). Any variety produced with genes from another species is technically a transgenic; it just does not count as a GMO because recombinant DNA was not used to move the gene from one species to the other.

Finally, the traditional concept in plant breeding has been that all plants have the same genes, and all that breeders have been doing is replacing the allele of one gene with another allele. The on-going, large-scale sequencing of plant genomes revealed that the traditional perspective is not completely correct, and different varieties of a crop differ by the presence and absence of hundreds, if not thousands, of genes (e.g., Agnieska et al., 2016; Hirsch et al., 2016). Thus breeders have not just been replacing alleles, they also have been inadvertently adding whole genes. The relevance is that genomes are clearly not adversely affected by the presence or absence of many genes, nor are there quantifiable safety issues associated with adding or removing genes.

**Figure 1:**
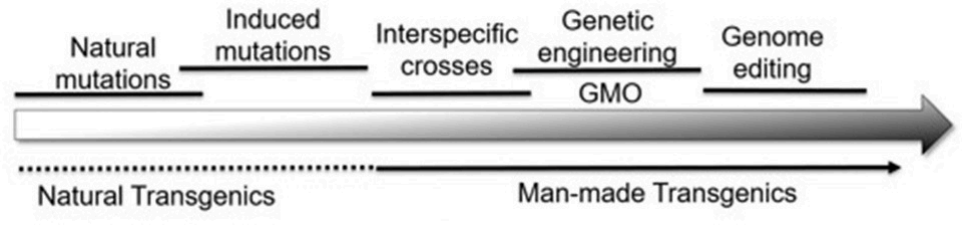
A wide range of natural and artificial processes serves to alter the plant genome in many ways. From a biological perspective, it becomes difficult, if not impossible, to draw vertical lines separating one category of modification from the other.

Collectively then, plant genomes are modified by a series of natural and artificial processes that result in the genetic variability used by breeders (Figure 1). With this background, it becomes possible to compare the changes at the DNA level that differentiate alleles from each other with those made by genome editing. Although the term “genome editing” implies a single process, editing can have three distinct effects on the plant genome:

1. A gene can be knocked out, i.e., inactivated by adding or deleting DNA, ranging from single base-pair deletions to deletion of the gene altogether.
2. A gene can be converted from one allele to another, by replacing base pairs from allele with base pairs from another allele.
3. A gene can be inserted in a predetermined place.

Looking at these three changes in more detail:

1. Gene knock-outs—the process creates non-functional alleles. As such there is nothing novel about non-functional alleles, which can be ubiquitous in plant populations. The changes at the DNA level made by editing are indistinguishable from those found naturally, which in turn are like those recapitulated in mutagenesis. In other words, a single-gene knock out from genome editing is indistinguishable from what happens naturally or in mutagenesis. The difference is that editing is more efficient at creating desired knockouts. Since natural and induced mutations take place at random locations, large numbers of plants must normally be screened to find one desired mutant. In contrast, genome editing can be targeted to a specific gene.
  However, few plant genes are found as single genes. Genes frequently are part of gene families. Alternatively, the plant can be an allopolyploid, which means the gene is duplicated on other chromosomes. The only way to get a recessive phenotype is if all gene copies are knocked out, and mutagenesis has never been effective at knocking out genes found in multiple copies (Stadler, 1929). In contrast, genome editing is adept at knocking out genes present in multiple copies. Thus, whenever a crop is found with multiple copies of the same gene knocked out, it will be almost certain that genome editing was used.
2. Converting one gene to another. Such editing recapitulates what breeders routinely do during backcrosses. The key difference is that breeders cannot replace single alleles with another in most species. Instead, they work with blocks of linked genes (Young and Tanksley, 1989). Therefore, genome editing can accomplish the task far more precisely and quickly than conventional breeding can ever do.
  On important difference is that some crop genes lie in low or non-recombinogenic regions of the chromosome. Thus, these genes have not been amenable to backcrossing during plant breeding programs. Genome editing ensures all genes are amenable to allele replacement.
3. Finally, there is site-specific gene insertion, a process that recapitulates the introduction of genes present in one variety but not another during conventional plant breeding. The difference, of course, is that in plant breeding the additional genes come from related species, while the genes can come from any organism when site-directed insertion is used. But then again, all plants are now known to have received genes from unrelated species (e.g., Bock, 2010).

All these considerations inform that genome editing simply creates the types of changes that are commonplace in nature. The main difference is that editing removes much of the randomness out of the process. Since risks always come from the final product and not from the way this product was obtained, there are no identifiable risks associated with editing that are different from those associated with conventional plant breeding, which in turn has a remarkable history of safe use (Steiner et al., 2013). The one exception would be if site directed insertion was to be used to insert a gene that codes for a toxin or an allergen, and procedures to evaluate the safety of novel genes are well established.

A final consideration is that genome editing can have off-target effects—it can create changes in the genome in places other than those intended. To evaluate the consequences of such off-target effects, it is once again necessary to compare genome editing with conventional plant breeding. The single largest source of off-target effects turns out to be conventional plant breeding (NASEM, 2004; Anderson et al., 2016). Likewise, traditional mutagenesis is rife with off-target effects that few people ever bother to detect or characterize. These historically have not been a cause for safety concerns, and the historical safe use of mutagenized crops bears witness to their safety. Thus, while it is possible to optimize the editing process to minimize off-target effects, and that these off-target effects would likely be removed during the subsequent breeding process, there is no reason to believe that any unintended edits left behind would pose a safety concern for crop plants.

In summary then, the unique features of genome editing are (1) its ability to edit genes present in multiple copies and (2) the ability to target the sites in the genome to be edited. At the DNA level, the changes are like those that take place naturally or in mutagenesis and that have a long history of safe use. The inescapable conclusion is that genome editing for gene knockouts and allele replacement must be considered to be at least as safe as conventional breeding. From the perspective of the FDA 1992 policy, edited plants would be subject to the same type of assessment as any traditionally bred variety. In other words, they should not need any special safety assessment.

**Figure 2:**
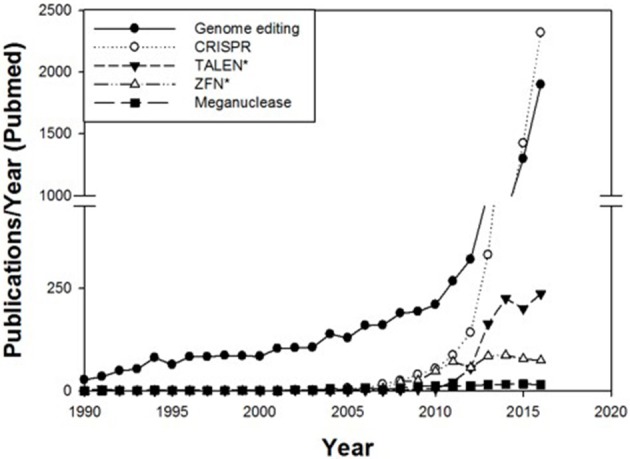
Number of publications per year in PubMed cited for the terms genome editing, CRISPR, TALEN*, ZFN* and meganucleases. The number of publications regarding these techniques has rapidly increased since 2008.

**Figure 3:**
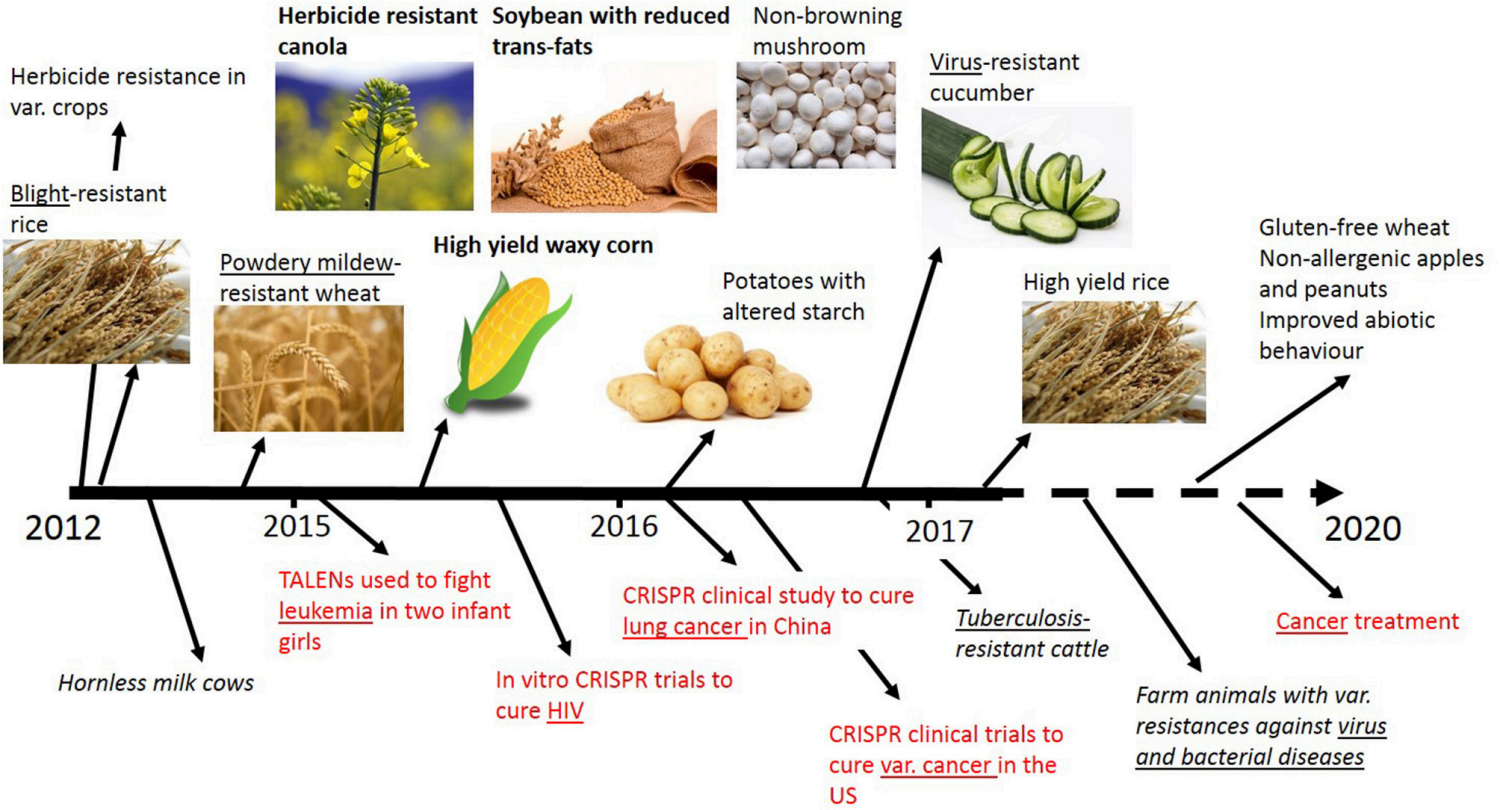
Timeline of selected traits modified by genome editing in plants, animals and for medical applications (red). Addressed diseases are underlined. Bold front: Applied for non-regulated status in the USA. No permissions were required for the use of the images^7^.

Thorben Sprink next described the regulatory challenges posed by genome-edited crops from the perspective of a public risk assessor in the EU:

In recent years genome editing and associated techniques have become a frequently used tool not only in research but also in applied breeding. Especially the CRISPR technology was a groundbreaking discovery, which is yet developed further with constantly expanding applications (Figure 2). Many traits in plant and animal breeding, as well as for medical application, have been addressed by genome editing and more are in progress (Figure 3). But only a handful of these have been subject to regulatory consideration in the US^5^ or in Europe (BVL)^6^.

The challenges of a regulatory framework in the face of new emerging technologies is not new to the European Commission. Back in late 2007, a working group was established to analyze a non-exhaustive list of techniques for which it is unclear whether or not they would result in GMO products under the current GMO regulation. The final report, however, has not been published and in the EU no final decisions have been made so far and no legal guidance has been published by the Commission (for a short summary of this topic, see Sprink et al., 2016).

Whether or not there are new environmental risk assessment (ERA) challenges that are associated with genome-edited crops has also been addressed by many scientific organizations. Their opinions and statements have been updated throughout the last 2 years. In 2017, the scientific advice mechanism (SAM) of the EU Commission has been requested to issue “*an explanatory note on new techniques in agricultural biotechnology including their potential agricultural application in synthetic biology and for gene drive, taking into consideration the most recent developments in the agricultural sector*” (SAM, 2016). This report has been published as of April 2017 (SAM, 2017). It compares NBTs with conventional breeding techniques (CBT) as well as with established techniques of genetic modification (ETGM) in seven categories: (i) Detectability/Identification, (ii) Unintended effects, (iii) Presence of foreign DNA, (iv) End product characteristics, (v) Ease of use/efficiency, (vi) Speed and costs and (vii) Maturity.

The SAM report points out that not only NBTs and ETGMs make use of genetic diversity and change to enable a genomic selection, but CBTs do so as well. They conclude that NBTs contain a variety technologies, and that, in some cases, the resulting products are comparable to the products of CBT as they do not contain foreign DNA, while in other cases they are comparable to products of ETGM, as NBTs also enable the use of foreign DNA. The report concludes that NBTs are more precise and result in lower amounts of unintended effects than CBT and ETGM do. Furthermore, especially genome editing techniques show a much lower number or a complete lack of unintended mutations as compared to products obtained via CBT, in particular when compared to mutation breeding or induced mutagenesis. Without prior knowledge of the alterations made to the genome by any of those techniques that do not introduce foreign DNA, the changes will be difficult to detect, and the identification of a particular technique as the cause of a certain alteration is impossible. The SAM declares that a safety assessment can only be made on a case by case basis depending on the traits of the end product or organism (SAM, 2017).

This statement echoes the updated statement of the European Plant Science Organisation (EPSO, 2017) which argued that “the EU regulatory framework for GMOs has become increasingly dysfunctional, as decisions are often not taken within the legal time frames, and often not on the basis of scientific evidence and risk assessment. The requested information and risk assessments are more comprehensive and are galvanized without scientific justification instead of being based on gained knowledge.” EPSO additionally calls attention to the point that GMOs should not merely be defined by the use of a certain technique but that a GMO also requires that a novel combination of genetic material beyond the natural borders of mating and recombination has been produced. This is not the case for point mutations obtained by genome editing (EPSO, 2017). Therefore, EPSO is in favor of a process- as well as product-based interpretation of the current framework of the EU and considering this to help to clarify the legal status of the NPBTs. EPSO supports the conclusions of the New Techniques Working Group, “that the legal definition of a GMO does not apply to most NPBTs and that these techniques either fall under the exemptions already established by the legislation or should be exempted as they do not differ from plants obtained by traditional breeding.”

The European Academies Science Advisory Council (EASAC) also updated their statement in March 2017 (EASAC, 2017). EASAC concludes that “*policy considerations should focus on the applications rather than on the genome editing procedure itself as an emerging technology*. *It should be ensured that regulation of applications is evidence based, takes into account likely benefits as well as hypothetical risks, and is proportionate and sufficiently flexible to cope future advances in the science.”* EASAC also focuses on the product as the trigger for regulation by asking EU regulators to “*confirm that the products of genome editing, when they do not contain DNA from an unrelated organism, do not fall within the scope of legislation on genetically modified organisms (GMOs)”*. Additionally, EASAC argues for “*a full transparency in disclosing the process used, but that the aim in the EU should be to regulate the specific agricultural trait/product rather than the technologies by which it is produced*.” This implies that the use of new technologies would be exempted from regulation if “*the genetic changes they produce are similar to, or indistinguishable from the product of conventional breeding, and if no novel, product-based risk is identified*.

Users of genome-editing technology for crop improvement face their own set of challenges that frame their perspective. Accordingly, Maria Fedorova gave a product developer perspective on genome editing and its similarities to and advantages over conventional breeding outcomes:

Traditional plant breeding has historically relied on plant's genetic variability to develop new varieties with improved characteristics. Favorable allelic variations, spontaneous mutations and induced random mutations have been a source of genetic diversity carried forward into commercially valuable genotypes. The ability to induce genetic variation in a targeted and more efficient fashion has been viewed as a much needed breakthrough and a challenge until recently. Genome editing, enabled by tools such as ZFN, TALEN or CRISPR/Cas, provides that breakthrough.

Genome editing can be defined as targeted modification of the plant's own genes without permanently introducing any foreign genetic material. This distinguishes genome-edited varieties from GMOs. Genome editing can produce plants indistinguishable from those that could arise from spontaneous or induced classical mutagenesis or be developed by introgression of the desired allele through a series of breeding crosses—i.e., tools deployed in conventional plant breeding.

CRISPR/Cas is one of the most recent genome editing tools, rapidly expanding its utility for academic research (reverse genetics, functional genomics studies) as well as practical application to develop new crop varieties with improved characteristics. CRISPR/Cas genome editing is viewed as a major advancement in precision plant breeding due to its versatility, efficiency and ability to work across species.

One of the examples of crop improvement using CRISPR/Cas genome editing is the next generation waxy (high amylopectin) maize, which was produced by targeted deletion of the waxy (*Wx1*) gene directly in elite inbred lines (Chilcoat et al., 2017). *Wx*1 is one of the most studied “classical” maize genes, with over 200 various spontaneous or induced mutations (deletions, insertions, translocations of various length) known to lead to the waxy phenotype (Wessler and Varagona, 1985; MaizeGDB, 2017). DuPont Pioneer's conventional waxy maize product, cultivated since the mid-1980s, is based on a spontaneous *Wx1* mutation (sequence deletion in the middle of the gene) from a maize variety discovered over 100 years ago (Fergason, 2001; Fan et al., 2009).

Limitations of the conventional waxy maize products are related to the introgression process of the *Wx1* mutation into top-performing modern elite lines and could be mitigated if the mutation in the *Wx1* gene was accomplished directly in elite inbred lines. Therefore, waxy maize elite inbred lines were generated by targeted *Wx1* mutation using CRISPR/Cas technology (Chilcoat et al., 2017). These lines exhibit the expected waxy phenotype, do not contain plasmid DNA used in the transformation process, and undergo extensive field evaluations according to common breeding practices.

It is fully appropriate to consider genome editing in the context of the range of plant breeding methods and, specifically, with the following two perspectives: how different is genome editing from processes occurring in nature or through conventional breeding methods? And, what is the likelihood of any given mutation to create a biosafety risk?

Inherent genetic variability is the biological mechanism allowing plants to adapt to ever-changing internal and external conditions. Genetic diversity is exceedingly common in plants, including important crop species such as maize, soybean, or rice (refer to Ching et al., 2002; Naito et al., 2006; Schnable et al., 2009; Springer et al., 2009; Parrott et al., 2012 for just a few examples). These spontaneously occurring processes are fundamental to crop evolution and the successful development of high performing elite varieties. To increase the genetic diversity, breeders can further boost the mutation rate by deploying classical (chemical, irradiation) mutagenesis tools, which generate multiple additional, random and unknown mutations besides the mutation of interest. As acknowledged by the European Food Safety Authority (EFSA), the frequency of mutations is predicted to be higher after classical mutational breeding (EFSA, 2012). With that, classical mutagenesis is broadly and successfully used in modern plant breeding, with over 3200 mutants registered in the FAO/EAEA mutant variety database^8^. Thus, the history of safe use of conventionally bred varieties demonstrates that a multitude of mutations occurring in a plant's own genes or intergenic sequences is unlikely to impact plant safety. The outstanding track record of conventional plant breeding provides a solid scientific basis for safety comparisons.

CRISPR/Cas genome editing allows to make many types of genetic changes similarly possible through conventional breeding but in a targeted fashion, i.e., in a more efficient, predictable and precise manner. The potential for off-target cutting can be mitigated by a variety of approaches, ranging from robust guide RNA design to modification of experimental conditions and to various molecular diagnostic tools tracing if an off-target cutting has actually occurred (Cameron et al., 2017 and references within; Svitashev et al., 2016). Furthermore, any potential off-target mutation, even if it occurred initially, would have been most likely segregated out during subsequent breeding cycles to develop the commercial variety. The generation of genome-edited plants without off-target mutations has been demonstrated in a number of publications (Baysal et al., 2016; Chandrasekaran et al., 2016; Nekrasov et al., 2017; Sánchez-León et al., 2017, to list a few).

Thus, risk assessment considerations associated with potential unintended effects or off-target cutting in genome-edited plants needs to be viewed in the context of the well-documented dynamic nature and plasticity of plant genomes. Similar to conventionally bred varieties, even if an off-target mutation were to occur, it is not expected to inherently make a genome-edited plant present a greater safety risk than a conventionally bred plant. The potential for unintended changes in the genome is not a unique feature of genome editing where any potential imprecision is expected to be significantly lower than the rates of spontaneous mutations or classical mutagenesis for which there is an established history of safe use.

Regulatory systems may face additional challenges posed by genome-edited products, which were discussed by Martin Lema. These include for instance the debate between technology-based and product-based regulations and the potential impact on product monitoring:

#### The core issue of regulatory touchstones

Debates regarding the regulatory status of genome-edited organisms generally follow a comparative approach with GMOs and with conventional organisms obtained by mutation and breeding. In general, these debates began considering *in extenso* technical aspects such as the possibility of generating the same kind of genetic modifications by other means, or the detectability of edited genes for the purpose of control and monitoring, or the relative safety of these products.

Certainly, these aspects are of high relevance. However, in the end regulators have to decide which regulation does or does not apply to a particular product. For this purpose, regulators need to resort to some legal “touchstones,” which most often are a definition (such as the GMO definition in most countries), triggers (such as “novel trait” in the Canadian regulatory system) or a list of inclusions/exclusions (like the Australian regulatory system).

Rules that determine whether or not an organism falls under a special GMO regulatory regime differ from one country to another. Quite often, their parameters for regulatory inclusion are based on product characteristics and/or the process used to obtain them. A recent review of the global GMO regulatory landscape which aims at anticipating the future scenario for genome-edited crops shows that the debate on “product-based” vs. “process-based” regulation is not the key influence when it comes to technology adoption (Ishii and Araki, 2017). The article also reports that many national regulations depart from the LMO definition of the Cartagena Protocol.^1^ which is worrying since the Protocol should act as a harmonizing factor. But while these diverging definitions have so far not created major issues for the classification of a plant variety as GMO (or comparable categories) or as a conventional crop, genome editing and other NBTs represent a broader spectrum of technical possibilities. The combination of this variety of technical possibilities with the wide array of subtle differences in national regulatory touchstones, may asymmetries that can affect trade.

#### First experiences in the regulation of genome-edited organisms

Debates on the regulatory status of genome-edited organisms (initially under the concept of “new breeding techniques”) date back at least to the year 2011 (Lusser et al., 2011). Nevertheless, to date there have been very few official regulatory determinations regarding these products (Wolt et al., 2016; Ishii and Araki, 2017). These articles and references therein provide a complete review of the first regulatory decisions in the USA, Canada, New Zealand and some isolated European countries, as well as the preliminary policymaking discussions in the European Union and some Asian countries. In addition to these reviews, the very latest developments for an updated account of the state of play are provided next.

Argentina has issued a working regulation that has been used effectively for the last 2 years in order to establish if specific products of genome editing are GMO or conventional crops (Whelan and Lema, 2015). Recently, this regulation was extended to genome-edited animals. Chile has issued a specific regulation in 2017 (SAG)^9^, and Brazil is in the process of issuing its own (CTNBIO)^10^. The three countries adopted quite similar technical criteria, both in terms of procedure (an *ex ante* assessment if the plant line is GM or not) and classification parameters (of which is paramount the presence or absence of r-DNA constructs in the genome of the line intended to be introduced in the market).

Israel also has recently issued a regulation, whose technical criteria resemble the ones applied in the Latin American countries mentioned before (Israel)^11^. Australia has launched a public consultation on GMO regulation amendments (OGTR)^12^. As a consequence of the proposed changes, some cases of genome editing may be exempted from regulation. However, the scope of potentially exempted products in Australia would be quite narrow compared to the approaches of the other countries that have made regulatory decisions until now.

**Figure 4:**
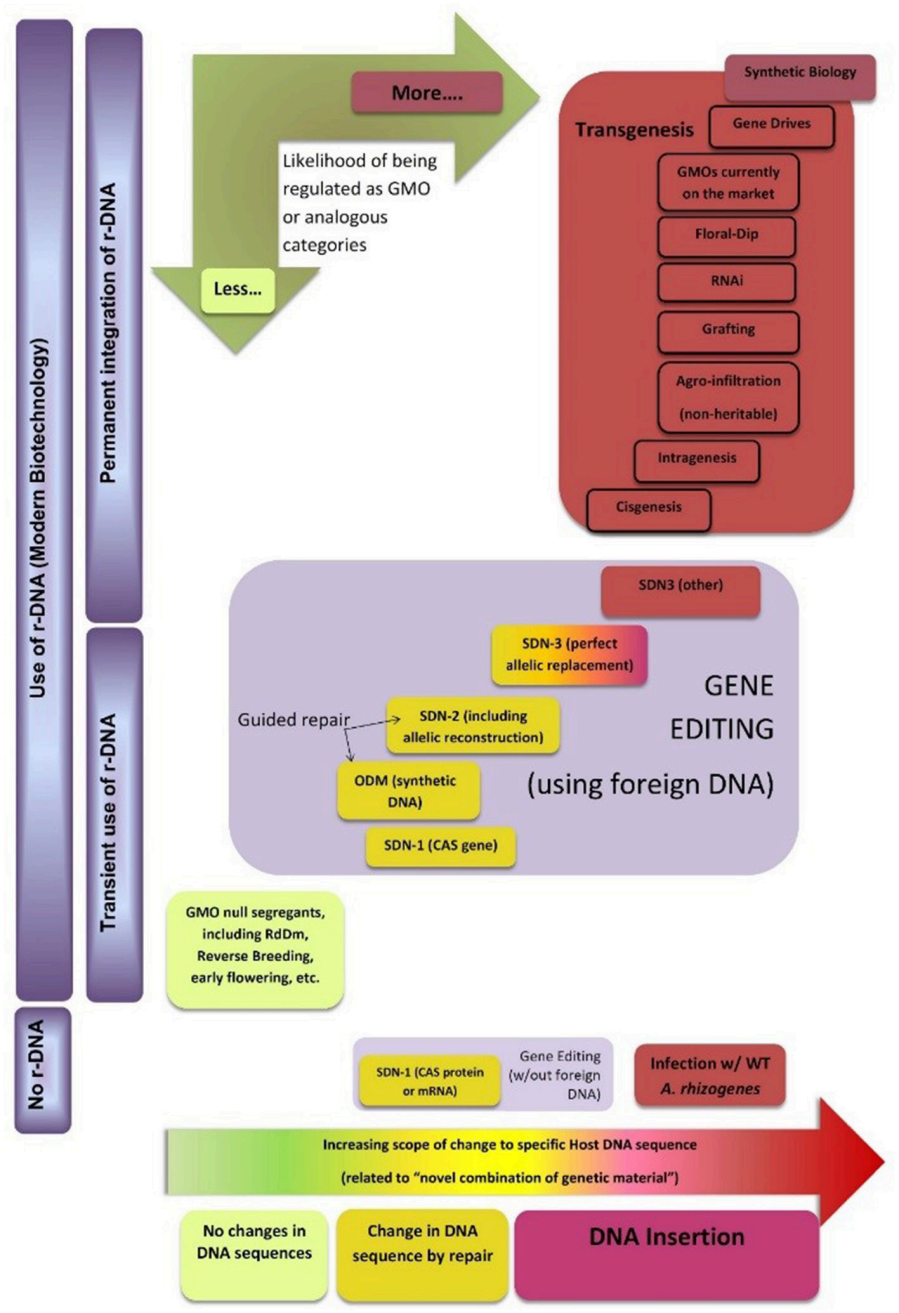
Classification map of new breeding techniques for regulatory purposes. The authors would like to thank Dr. Huw Dylan Jones from Aberystwyth University for fruitful discussion during the elaboration of this diagram. See text for details.

Figure 4 shows a possible classification map of new breeding techniques, including genome editing, for regulatory purposes (the horizontal dimension indicates an increasing degree of intervention in specific DNA sequences of the plant that are allowed by each technology). This does not correspond to any country in particular but tries to capture emerging similarities in how the techniques seem to be perceived in different regulatory environments. It is based on the initial decisions or *ad hoc* regulations issued by some governments, as well as advice by official scientific bodies of other governments. As most countries worldwide are members of the Cartagena Protocol or use its LMO definition as a definition for GMO, its main concepts are also incorporated into the conceptual map. For practical purposes this definition encompasses two main requirements: The first one is for the organism to have a novel combination of genetic material, which can be related to the horizontal dimension as described. The second one is the use of recombinant DNA (r-DNA) to obtain such novel combination. Therefore, the map uses the vertical dimension to separate techniques that do not use r-DNA from those that use it transiently (but where it can be removed from the final organism) and those where r-DNA is permanently incorporated into the recipient genome.

As mentioned, most countries in the world regulate “GMO.” Therefore, we have used this term in a wind rose incorporated to the map to indicate the likelihood with which products derived from these technologies may fall under a special regulation. The conceptual map does not include a regulatory boundary because the issue is far from being harmonized. However, it may help experts and policymakers from different countries to identify common grounds and pinpoint where exactly their differences in criteria are located, thus supporting harmonization efforts.

#### Product monitoring

It has been argued that the detectability of genome edited products is technically harder compared to GMOs, and that therefore there is no point in having them regulated. In terms of policymaking, this argument is moot. Products are regulated because sectors of society want them to be regulated. If there are technical tools to detect the product the better, but if not, regulation can also be based on a system of sworn statements, traceability, etc.

For most products of genome editing, there is a clear signature in the DNA, for instance the exact stretch of nucleotides erased. If that signature is revealed by the developer, the same PCR technology used for detecting GMOs can be applied to the detection and monitoring of genome-edited products in most cases.

Conversely, there are some concerns over the possibility of new lines or breeds for which the developer does not correctly indicate the technique by which they were obtained, since (in some cases) identical changes in the DNA sequence can be generated by either genome editing or conventional breeding. Of course, detectability in this hypothetical case is more difficult, but this is also true for a genetically modified (GM) product. However, this scenario is particularly unlikely (both for GM and genome-edited organisms) as the developer would be trapped in the prisoner's dilemma because of the possibility of being “betrayed” by information released by employees, collaborators, technical publications, etc. In summary, the detectability of genome-edited products that might reach the market is not significantly different from that of GMOs and therefore, if necessary, would be covered by the already existing international instruments and technical tools.

There is certainly potential for a rugged landscape in the regulation of products from genome editing. This landscape could lead to asymmetries in the regulatory/approval status in different countries, and contribute to the “low level presence” trade issues currently experienced with GM crops (OECD, 2013). In such scenario, detection methods and other monitoring measures currently applied to GM crops will likely play the same roles and with the same efficacy in the case of gene-edited crops. The infrastructure for such monitoring and detection is already in place in many countries.

#### Social issues

From the viewpoint of sociology of science and technology, genome-edited products can be regarded to be in a state of “interpretative flexibility,” leaving room for discussion on whether or not they are GMO. This also means that a list of changing actors are molding the issue with evolving alliances and changing interests, and that the matter is far from being stabilized at the conceptual level.

Clearly, finding the adequate regulatory approach not only entails subjects pertaining to safety information and legal definitions; it also interplays with socio-technical resistance, international trade and innovation in agriculture. Therefore, even when the official scientific advisory bodies may advice that at least some genome-edited products should be regarded as conventional breeds or varieties from a regulatory standpoint, the political decision makers may decide otherwise for various reasons.

A relevant example of political authorities not following official scientific advice was the moratorium for GM crops in the European Union which led to a dispute in the WTO over the validity of such moratorium as a sanitary measure (Disdier and Fontagné, 2010). During the case, the European Community Authorities tried to discredit the advice provided by the European Food Safety Authority (EFSA), which asserted that products were safe. This strategy aimed at justifying the governmental moratorium in GMO approvals as a sanitary measure. Finally, the work of EFSA was proven to be based on sound science using internationally agreed standards, such as the *Codex Alimentarius* Guidelines for biotechnology products (CODEX)^13^.

Political decisions might result in some genome-edited products being “over”-regulated, contrary to scientific advice. It has been warned that such decisions would hamper innovation in agriculture, with potential impacts on economy and sustainability (Jones, 2015). This warning has been raised repeatedly by representatives of the academic, seed, and breeding sectors. However, these opinions have been mostly of unsubstantiated and qualitative nature.

Accordingly, as discussed in a recent article (Whelan and Lema, 2017) decision makers may need formal and quantitative studies on potential economic impacts of handling genome-edited products under different regulatory scenarios. Such studies would allow them to weigh the impact of different regulatory/policymaking options on the economy (considering trade, agroindustrial innovation and productivity). A formal analysis of the trajectory or dynamics that the interpretative flexibility is taking may be useful to anticipate the social perception of these decisions.

#### Private regulations

Interestingly, as the list of social actors increases, the interpretative flexibility extends to aspects beyond sanitary regulations. For instance in Argentina and other countries there are projects applying genome editing to sport animals, such as race dogs and polo horses. This has initiated a debate in the corresponding breeding or sport associations as to whether the use of genome editing may be anti-sportive, such as unfair play or gene doping (AACCP^14^; Oliveira et al., 2011; Reuters^15^).

In conclusion, regulators and policymakers have become familiar with the technical aspects of genome editing, and debates on their appropriate regulation have sparked worldwide. These debates have extended over several years, and a wide range of actors are already involved. Some issues included in the debates, such as “product-based” vs. “process-based” regulation or product detectability initially seemed very relevant, but are not actually contributing much to decision-making. It is important to clarify that such technical debates are useful only if they help decide how to interpret and/or modify the regulatory touchstones of each country.

To date, some nations with a significant participation in international trade have already established their criteria or are close to do so. At this stage, the true remaining challenges for establishing a sound and globally harmonized regulation are more of a social than technical nature; therefore, they include an appropriate assessment of the implications of regulatory alternatives upon issues such as social perception, international trade, local innovation, and competitiveness of agroindustrial chains.

Finally, Jeffrey Wolt provided perspectives on the National Academies of Sciences, Engineering and Medicine (US-NASEM) report, which takes a step toward preparing for future biotechnology products:

In 2015, a White House Memorandum called for modernization of the biotechnology regulatory system with a focus on updating the Coordinated Framework for Biotechnology (EOP, 2015). The intent of this action was to “*clarify the roles and responsibilities of the agencies that regulate to ‘products of biotechnology';*” to formulate long-term strategy for biotechnology regulatory system to efficiently assess risks “*associated with future products of biotechnology;*” to support innovation, protect health and environment, promote public confidence in regulatory process, increase transparency and predictability, and reduce unnecessary costs and burdens. The memo additionally specified “*commissioning of an external, independent analysis of the future landscape of biotechnology products*” with a focus on potential new risks and risk assessment frameworks for biotechnology products expected to emerge in the marketplace in the next 5–10 years. This effort was initiated by the Office of Science and Technology Policy (OSTP) in July 2015 and the task was undertaken by a committee of science and policy experts, convened through the U.S. National Academies of Science, Engineering and Medicine, which produced the report *Preparing for Future Products of Biotechnology* (NASEM, 2017). The committee's deliberations reflect recognition of rapid growth in the bioeconomy and the need for the U.S. regulatory system to keep pace. Their findings align with those of the U.S. Office of Science and Technology Policy's internal analysis reflecting a modernized regulatory system that effectively anticipates and addresses emerging products of biotechnology.

This contribution gives a brief synopsis of the report and introduces the implications to the emerging use of plant genome editing for crop improvement and how this may impact the ecological risk assessment process as well as regulation of future products of biotechnology.

While the report says little specifically with regard to genome-edited crops (in the view of the committee crops derived by genome editing were an existing reality for the U.S. regulatory system so represent current rather than future biotechnology products) here, a perspective as to how risk and regulatory considerations for genome-edited crops will influence the ability for innovative new biotechnologies to enter the marketplace will be provided.

#### Background on the report

The rapidly changing field of biotechnology has led to innovations that were unanticipated at the time the Coordinated Framework for Biotechnology was first developed. The scope of revision of the Coordinated Framework is to address products of biotechnology more broadly and therefore, *Preparing for Future Products of Biotechnology* (NASEM, 2017) considers for its purposes “*products developed through genetic engineering or [genome engineering or] the targeted or in vitro manipulation of genetic information of organisms, including plants, animals, and microbes*.” The report's key themes recognize that: (1) The “*bioeconomy is growing rapidly and the U.S. regulatory system needs to provide a balanced approach for consideration of the many competing interests in the face of this expansion*.” (2) A “*profusion of biotechnology products [envisioned] over the next 5–10 years has the potential to overwhelm the U.S. regulatory system*.” (3) Regulators will face difficult challenges that go beyond considerations of contained industrial uses and traditional environmental release as the “*safe use of new biotechnology products [will require] rigorous, predictable, and transparent risk-analysis processes that mirror the scope, scale, complexity, and tempo of biotechnology development. (4) Agencies involved in regulation of future biotechnology products would benefit from adopting recommendations made by previous National Academies' committees*.”

The urgency for a revised Coordinated Framework to address the rapidly emerging bioeconomy is evidenced in accelerants that are hastening bioengineering innovation and product development. But future products of the bioeconomy are not envisioned to reflect new risk-assessment endpoints for ecological risk assessment (ERA) and regulatory consideration; rather these products represent differing and high complexity pathways to those endpoints. Significant increases in the rate, number, and complexity of biotechnology products, and the diversity of actors involved in the research and development process, will challenge the abilities of Federal agencies. Enabling effective regulation will require streamlined access to the regulatory system in a manner which is highly transparent to developers and the public alike.

In the view of the committee (NASEM, 2017), the current Coordinated Framework for Regulation of Biotechnology appears to have considerable flexibility to address these challenges, but jurisdictional considerations have the potential to duplicate the regulatory effort or leave gaps in regulatory oversight. The U.S. biotechnology regulatory system is complex and fragmented and can be difficult to navigate. This complexity causes uncertainty and a lack of predictability for developers of future biotechnology products, which in turn has the potential for loss of public confidence in regulation of future biotechnology products. Therefore, a more streamlined, flexible and transparent system is needed.

The report concluded that U.S. “Agencies involved in regulation of future biotechnology products should increase scientific capabilities, tools, expertise, and horizon scanning in key areas of expected growth of biotechnology, including natural, regulatory, and social sciences.” Additionally, pilot projects may be useful “to advance understanding and use of risk assessments and benefit analyses for future biotechnology products that are unfamiliar and complex.” And finally, “agencies that fund biotechnology research with the potential to lead to new biotechnology products should increase their investments in regulatory science and link research and education activities to regulatory-science activities” (NASEM, 2017).

#### Perspectives relative to era and regulation of genome edited crops

Plant genome editing is on the leading edge of massive innovation in the field of bioengineering, which will result in diverse product types that have not been previously considered within a formal regulatory context. Assessment strategies and regulatory approaches for genome-edited crops will establish the paradigm for innovation that follows. Using approaches established for transgenic crops may hobble the abilities of regulatory authorities with knock-on effects to innovation for the bioeconomy. Therefore, there is a need to streamline and increase collaboration amongst regulatory authorities, to triage risk assessments to focus on novel/complex products, and to adopt extra-regulatory approaches to governance where appropriate.

The increasingly novel and complex products and product uses released to consumers and the environment will be difficult to monitor and recall. For instance, many genome-edited crops will be indistinguishable from varieties developed through traditional selective plant breeding, and therefore a more focused consideration of the phenotype intended for deployment will be of greater concern that the process that has been used. Enhancing capacity and capabilities for regulatory science education of scientists and of active and engaged publics will be needed. In addition, extra-regulatory research governance mechanisms should be encouraged to identify and manage risks and uncertainties earlier in the research and development process. For instance, many public institutions in the U.S. have already instituted processes within institutional biosafety committees to ensure that appropriate stepwise assessments and confinement actions are made to limit the possibility for the initiation and deployment of unintended gene drives as a result of genome editing. These and related actions can appropriately broaden the parties responsible for biosafety to encompass researchers and public parties in addition to the regulated community and regulators.

## World café—interactive session on novel features to consider

All the topics, challenges and perspectives that have been presented in the expert contributions provided a base for an interactive session, a “World Café” focused on novel features to consider that may result from the application of genome editing in plant breeding. In this session, which was led by Nina Duensing, Thorben Sprink, and Detlef Bartsch, the participants had the opportunity and were strongly encouraged to discuss some key questions of three different aspects regarding the risk assessment, monitoring and regulation of genome-edited plants. Each discussion group of approximately 20 participants rotated through all three topics, prioritizing their own and the previous groups' arguments.

### Environmental risk assessment—novel demands?

**Figure 5:**
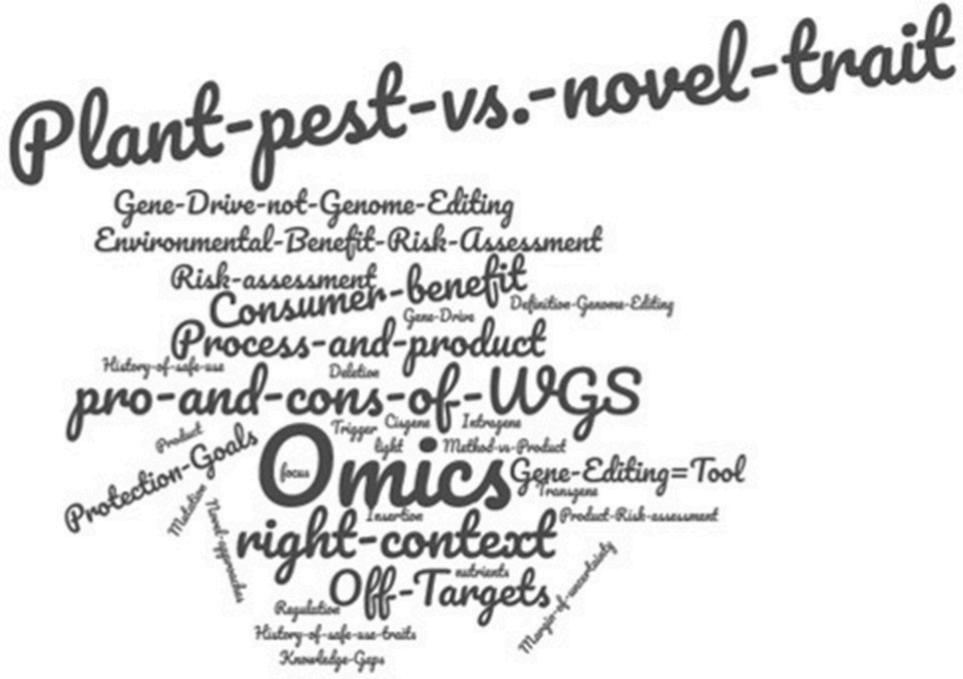
World Café key contributions for the topic “*Environmental Risk Assessment - novel demands?”* Letter size represents the participants' ranking according importance.

In this topic, questions regarding the Environmental Risk Assessment (ERA) of genome-edited plants were discussed: Are there novel demands for the ERA of genome-edited organisms? Are there additional, novel risks to be considered, or is a simplified procedure, an “ERA light,” possible? Is there a correlation of a potential risk with the modification process itself, or rather with the introduced traits? Are there potential knowledge gaps, for example the probability of additional, unintended changes (“off-target effects”)? An overview of the key contributions is provided in Figure 5.

The most important consensus of the participants was that a combined process- and product-based approach was crucial and that all risk assessment should regard the used techniques only in the context to the modified trait. According to the majority of participants this is also true with regard to the concept of “history of safe use” which needs to be considered when performing a comparative risk assessment, as higher precision and a much lesser frequency of unintended changes are to be expected from genome editing applications. Therefore, the concept of “history of safe use” should be applied focusing on the characteristics of the resulting crop plant, not the technique used to generate them. It was reiterated that genome editing itself is only a tool; for environmental assessment, the characteristics of the final organism are decisive, not the tools that were applied to generate them.

Additionally, it became clear in the course of the discussion that the classification of genome editing applications in “site-directed nuclease” (SDN)-1, SDN-2 and SDN-3 is not generally or comprehensively defined, yet. ODM, SDN-1 and−2 are broadly seen as a targeted form of mutagenesis. Products resulting from SDN-3 are seen as GMOs, but less data may be required for their risk-assessment of cisgenic or intragenic plants than for classical transgenic plants. Furthermore, the World Café initiated a discussion on whether genome-edited plants possess specific risks for generally agreed protection goals. The general opinion was that there are no specific threats initiated by genome editing techniques. Here, again, it is crucial to restate that genome editing techniques are tools and, again, a potential risk of a plant is defined by its traits, not by the technique used in the breeding process.

Whole genome sequencing (WGS) and “-omics” tools have been a point of discussion but were generally considered of minor importance for the risk assessment. Also, gene drive applications were generally seen as less relevant for plants and for use in agricultural systems. However, noticeably, not all participants regarded gene drive systems as genome editing *per se*, and, very accurately, organisms containing engineered gene drives were generally regarded as GMOs, as these applications require the introduction of a foreign gene (i.e., Cas9). Their Regulation, risk assessment and monitoring would therefore already be coverd by GMO regulatory requirements. However, other participants mentioned that natural occurring gene drive systems, such as Medea, are already present in populations.

The World Café organizers' final conclusion of this session was that there are, in principle, no demands for a novel ERA as the existing regulatory frameworks would cover all genome-edited organisms. Instead, the improved precision plus lower probability of off-target effects as compared to conventional methods would rather simplify and focus any ERA on the introduced trait. Therefore, the adjustment of current frameworks to the increased technical precision seems appropriate.

### Monitoring—detection and identification of new products after placing on the market

**Figure 6:**
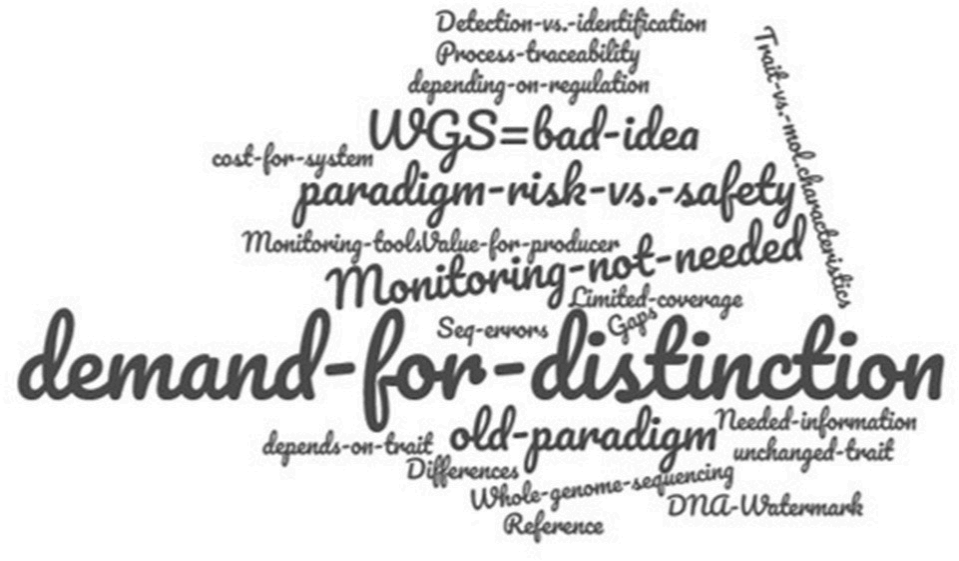
World Café key contributions for the topic “*Monitoring - detection and identification of new products after placing on the market.”* Letter size represents the participants' ranking according importance.

In case genome-edited organisms are to be classified as GMOs and therefore be subject to GMO regulation, a monitoring system will be required for their authorization in some jurisdictions. Such a system needs specific detection and identification methods in place. Therefore, in this topics challenges and limits regarding the detection and monitoring of genome-edited organisms as well as whether or not there is a need for their detection, was discussed An overview of the key contributions to this topic is provided in Figure 6.

Intriguingly, there was some controversy on whether or not genome-edited organisms are, in all cases, detectable and unambiguously identifiable as such. Molecularly, small nucleotide replacements, insertions or deletions are identical, whether they occurred spontaneously, were induced by classical mutagenesis or site-specifically introduced via genome editing. Therefore, unless a foreign DNA (originating from a non-crossable, sexually incompatible organism) is inserted into a given genome, the resulting organisms will be indistinguishable from those that were developed using traditional breeding techniques. So, how shall they be monitored?

Due to the high rate of spontaneously occurring mutations and the inherent error rate of WGS applications, this tool was not considered to be an appropriate method for detection and identification of genome-edited plants. Sequence differences are expected even between close relatives or direct offspring, therefore a detected difference in any genome sequence as compared to the chosen reference genome can impossibly be attributed to a previous genome editing application. It could as well have occurred naturally or be attributed to a sequencing artifact.

It was recognized, though, that the breeders and developers themselves will have an interest for their products to be distinguishable from others. And as long as the information on the modification is provided, the modification is detectable using standard molecular biology tools, enabling an identification of the modified organism. An unknown, undisclosed modification which does not involve the incorporation of foreign sequences, however, will be hard to detect; and even if it was detected, identifying how it was introduced, i.e., by targeted mutation using genome editing tools, conventional breeding, including random mutagenesis, or naturally occurring mutations, is impossible.

While it was recognized by the participants that some form of control of genome-edited organisms seems to be desired by sectors of the public, scientifically a specific monitoring of such organisms is regarded as unnecessary, mostly because in comparison to conventional applications, a higher level of precision and safety are to be expected from genome editing. There was a high level of consensus—especially within participants from Central and South America—that the subject of detection and identification of crop plants that were produced by genome editing techniques was of minor relevance as these organisms and products thereof must not be defined as GMOs and therefore are not required to be detected or identified. The fact that it might, nonetheless, be possible that in some countries these crops might be classified as GMOs was met with incomprehension or even reluctance. This reflected the broad concurrence that the EU approach toward genome editing and other precision breeding innovations was overly restrictive and over-regulating.

The World Café organizers' final conclusion of this session was that there is no reason to establish a specific regulatory monitoring for genome-edited plants that are indistinguishable from those that were developed using traditional breeding techniques. Not only would specific monitoring requirements for basically identical varieties be scientifically unreasonable, but to require the detection and identification of such single or few nucleotide-edited organisms or products thereof would also imply an almost unsurmountable challenge to (official) analytic laboratories and enforcement institutions. In addition, the requirement of cost-effectiveness is hardly to be met even if new traceability chains would be established.

### Harmonization of regulation

**Figure 7:**
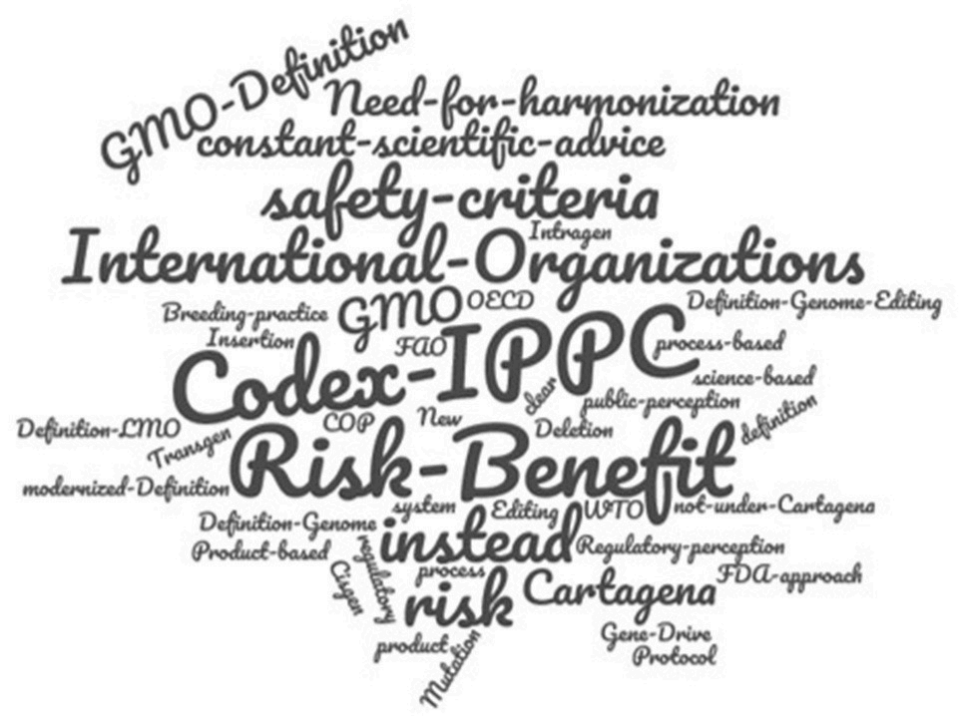
World Café key contributions for the topic “*Harmonization of regulation*.” Letter size represents the participants' ranking according importance.

Free global trade requires internationally harmonized regulations. Different GMO definitions and therefore different regulation and authorization requirements would hinder international exchange—especially if the products are indistinguishable. To which extent will an international harmonization of regulation requirements be possible? Which organizations are to be responsible and able to advance and coordinate a harmonization process? Will an international consensus for regulation be possible? And if so, which consensus is favored? An overview of the key contributions is provided in Figure 7.

The most crucial point here was the relevance of a science-based risk and benefit analysis in order to increase public awareness and the awareness of the regulatory authorities. Various organizations' tasks and responsibilities in forwarding regulatory harmonization efforts were discussed: The integration of genome editing into the *Codex Alimentarius*^10^ was considered as the most internationally useful way to provide a collection of standards for a harmonization. In contrast, the Cartagena Protocol on Biosafety to the Convention on Biological Diversity was not considered as a useful tool to advance harmonization. This sentiment may be attributed to the scope of the Cartagena Protocol which is the safe handling, transport and use of GMOs. The predominant reasoning within the vast majority of discussion participants was that organisms resulting from genome editing applications (excluding those that involve the integration of foreign DNA into the recipient's genome) are not GMOs.

Another consideration was the need to reconsider the wording of legislation documents: Instead of focusing on “risks,” “safety criteria” should be the focus. The GMO definition is of central importance for an international harmonization of the regulation of genome-edited organisms. The majority of the workshop participants clearly were in favor of a process- and product-based approach, and are also in favor of an open and transparent process leading toward international coordination.

The World Café organizers' final conclusion of this session was that an international harmonization of regulation requirements is possible and urgently needed to close the risk-benefit gap between precaution and innovation potential of new genome edited organisms. It will have to be determined, which international organization can best take on this task, but failing an international harmonization will almost inevitably lead to insecurities and trade limitations. There is a need for a clear, harmonized GMO definition and for a science-based analysis not focusing merely on the potential risk but also on the benefits of the application of newly emerging biotechnology applications, including genome editing.

### World café: summary and conclusion

In the overwhelming majority of the 38 countries represented at the symposium, and supposedly also represented to a large extend by the workshop participants in this session (approximately 60 participants), the competent authorities pursue both process- and product-based approaches for the evaluation of genome editing and the resulting products. Given the increased efficiency and precision of these techniques, a comparably higher safety for humans, animals, and the environment is to be expected from genome-edited organisms. In many cases, a genome-edited crop variety will be indistinguishable from a variety that was developed using conventional breeding techniques. While developers might use genome editing applications to improve a plant's traits or characteristics by targeted mutagenesis, molecularly that modification in the DNA sequence will not differ from a mutation that has occurred naturally or through conventional mutagenesis. Therefore, a general classification as GMO (under the current GMO definition), including all regulatory, detection and monitoring requirements, is not desired and not seen as scientifically justified or practically enforceable.

A globally harmonized regulatory approach is considered highly important and might, in principle, enable the linkage of innovation and precaution. There was a general agreement that products resulting from genome editing will reach the market, and to date some crop plant varieties are already being commercially produced in the U.S. and a few other countries. A potentially emerging solely product-based regulation in some countries may cause trade issues, not only for genome-edited crops but also for conventional breeding products.

There was a debate of whether a new regulatory framework is needed for products resulting from genome editing or if the already existing frameworks are adequate. If a novel framework was needed, could there be a science-based risk assessment for genome-edited products? How could such a risk assessment look like? What should be included? These questions were intensely discussed and further points of contention which will have to be addressed in the future were identified: To date, a broadly accepted definition of what is considered “natural” in a regulatory context is missing or inconsistent, and a definition of “recombinant nucleic acid” is lacking, leaving spaces for interpretation. There is also a dissent in the possible regulation of products resulting from SDN-3 approaches using self-cloning and whether or not it is possible to detect and identify the products of genome editing solely by the product itself.

The World Café organizers' final conclusions were that no new regulatory frameworks for genome-edited plants are considered necessary. Existing frameworks are still adequate but may need adjustments, for example concerning a decrease in data requirements due to the increase in precision, and if comparability with already existing—safely used—non-GM and GM organisms allows this. Also, if a genome-edited plant is indistinguishable from a variety that was developed using traditional breeding techniques there is no scientific reason to call for a specific regulatory monitoring of this plant. Finally, international regulation should allow for a flexible handling of constantly emerging scientific progress. This requires an internationally harmonized GMO definition and a flexible adaptation of regulation to technological progress, taking into account appropriate scientific supervision.

## Conclusion

Increasing technical efficacy and decreasing costs revolutionize the tools that science-driven economies can apply to increase a crop's genetic variability, a major resource for plant breeding. This high efficacy and low cost, however, could be rendered useless if appropriate regulation is not established to provide a framework that enables the use of these new tools. It is time and opportunity to find the right balance between precaution and innovation for the benefit of plant breeding. Risk assessment and regulation need to balance the public's need for food, feed, and environmental safety with the costs for developers, growers, shippers and processers without wasting resources and in a proportionate, science-based way. This requires an international harmonization of regulatory frameworks, and while there is currently no demand for a novel ERA for genome-edited organisms, adapting the existing frameworks to the increased technical precision as compared to conventional methods seems appropriate.

## Disclaimer

The information and views are those of the authors as individuals and experts in the field and do not necessarily represent those of the organizations they work for.

## Author contributions

ND, TS, and DB wrote, checked and edited the manuscript. WP, TS, MF, ML, and JW contributed the sections on their respective talks. ND, TS, and DB contributed the sections on the interactive session. All authors have read and approved the manuscript for publication.

### Conflict of interest statement

MF is an employee of Corteva Agriscience™, Agriculture Division of DowDuPont™, a leader in CRISPR/Cas advanced breeding applications in agriculture and is developing its first CRISPR/Cas enabled commercial products. The remaining authors declare that the research was conducted in the absence of any commercial or financial relationships that could be construed as a potential conflict of interest.

## Footnotes

^1^USDA (2017) *Press. Release No. 0144.17*.

^2^USDA (2018) *Details on USDA Plant Breeding Innovations*. https://www.aphis.usda.gov/aphis/ourfocus/biotechnology/brs-news-and-information/pbi-details

^3^The Cartagena Protocol on Biosafety to the Convention on Biological Diversity defines a so-called living modified organism (LMO) in Article 3 (g) as “any living organism that possesses a novel combination of genetic material obtained through the use of modern biotechnology.” Modern biotechnology is further defined in Article 3 (i) as “the application of: (a) *In vitro* nucleic acid techniques, including recombinant deoxyribonucleic acid (DNA) and direct injection of nucleic acid into cells or organelles, or (b) Fusion of cells beyond the taxonomic family, that overcome natural physiological reproductive or recombination barriers and that are not techniques used in traditional breeding and selection.”

^4^http://isbr.info/ISBGMO14

^5^https://www.aphis.usda.gov/aphis/ourfocus/biotechnology/am-i-regulated/Regulated_Article_Letters_of_Inquiry

^6^https://www.bvl.bund.de/DE/06_Gentechnik/04_Fachmeldungen/2015/2015_06_03_Fa_CIBUS.html)

^7^https://pixabay.com/de/reis-und-mais-reis-mais-sorte-korn-587593/; https://pxhere.com/de/photo/707286; https://pixabay.com/de/mais-ernte-gem%C3%BCse-lebensmittel-152037/; https://pixabay.com/de/raps-landwirtschaft-feld-bl%C3%BCte-1333511/; https://pixabay.com/de/sojabohnen-pflanzen-saatgut-tasche-2039639/; https://pixabay.com/de/pilze-champignons-wei%C3%9F-1351060/; https://pixabay.com/de/kartoffeln-erd%C3%A4pfel-ungesch%C3%A4lt-2829118/; https://pixabay.com/de/gurke-salat-lebensmittel-gesund-685704/

^8^https://mvd.iaea.org/

^9^SAG 82017. http://www.sag.cl/sites/default/files/RES_1523_2001.pdf

^10^CTNBIO (2018) Resolution no.16 of January 15 2018.; Brazilian Official Gazette No.15, section 1, pages 7–8. (Published January 22 2018)

^11^Israel, 2017. Summary of National Committee for Transgenic Plants Meeting of August 8 2016 (March 2017, not publicly available, but covered e.g., here: http://news.agropages.com/News/NewsDetail-−22144.htm)

^12^OGTR, 2017. http://www.ogtr.gov.au/internet/ogtr/publishing.nsf/Content/reviewregulations-1

^13^CODEX, 2017. http://www.fao.org/fao-who-codexalimentarius/thematic-areas/biotechnology/en/

^14^AACCP, 2017. http://www.criapoloargentino.com.ar/?sec=7&nota=965

^15^Reuters, 2007. https://uk.reuters.com/article/science-genes-dogs-dc/gene-makes-racing-dogs-fast-study-finds-idUKN0118454720070501
